# Association Between Living Risk and Healthy Life Years Lost Due to Multimorbidity: Observations From the China Health and Retirement Longitudinal Study

**DOI:** 10.3389/fmed.2022.831544

**Published:** 2022-03-17

**Authors:** Xinlei Miao, Jun Chen, Wen Meng, Qiong Wu, Zhiyuan Wu, Lin Ren, Yue Cai, Xiuhua Guo, Xiang Zhang, Qun Meng

**Affiliations:** ^1^School of Public Health, Capital Medical University, Beijing, China; ^2^Beijing Municipal Key Laboratory of Clinical Epidemiology, Beijing, China; ^3^Center of Information Statistic, Health Information Center of Shaanxi Province, Xi'an, China; ^4^Chinese Medical Doctor Association, Beijing, China; ^5^Center of Big Data Office, National Health Commission of the People's Republic of China, Beijing, China; ^6^Center of Information Statistic, National Health Commission of the People's Republic of China, Beijing, China; ^7^Information Management Center, Affiliated Zhongshan Hospital of Dalian University, Dalian, China; ^8^Comprehensive Supervision Bureau, National Health Commission of the People's Republic of China, Beijing, China

**Keywords:** multimorbidity, healthy years of life lost, living risk, quality of life, health-related factors

## Abstract

**Background:**

Multimorbidity has an effect on life expectancy, while its effect on healthy life years is unclear. This study aims to investigate the associations between healthy life years lost due to multimorbidity and living risk.

**Methods:**

The participants of The China Health and Retirement Longitudinal Study (CHARLS) were assessed at four visits between 2011 (baseline) and 2018. At baseline, 13,949 individuals were administered surveys. A combined score based on seven health-related factors was calculated, and the participants were classified into 3 groups based on living risk. We used the adjusted Cox regression methods to examine the associations between living risk groups and multimorbidity. We estimated the healthy life years lost due to multimorbidity using the Sullivan method.

**Results:**

A total of 9,091 adults aged 45 years or older (mean age of 59.55 ± 9.50 years with one disease, 52.60% women) were analyzed in the CHARLS. The probability of no multimorbidity over 7 years decreased from 0.9947 to 0.9697 in the low-risk group, whereas the probability of multimorbidity in low living risk was lower than that of high living risk, ranging from HR 1.253 (95% CI.992–1.581; *P* = 0.058) to 1.431 (1.05–1.949; *P* = 0.023) in sex, and ranging from HR 1.340 (95% CI 1.106–1.623; *P* = 0.003) to 2.002 (1.058–3.787; *P* = 0.033) in area. At 45 years, the healthy life years lost in men was <0.27 years compared to women in the low-risk group. Hypertension increased the risk of multimorbidity with an HR of 1.5 (95% CI 1.21–1.91; *P* < 0.001) in men. In urban areas, participants with diabetes had 3.2 times (95% CI 1.75–5.94, *P* < 0.001) higher risk of multimorbidity than participants without diabetes.

**Conclusions:**

These findings indicate that a low-risk lifestyle could decrease the loss of healthy life years under multimorbidity. The probability of multimorbidity in women and in urban areas was high. Hypertension was correlated with the hazard risk of multimorbidity.

## Introduction

Population aging has become a serious issue of global concern. Epidemiological studies have shown that the number and proportion of people aged 60 years and older in the population is increasing, and it is expected to increase to 2.1 billion in 2050 (1). Longer life is a valuable resource, while the opportunities that come with these additional years are largely determined by healthy years. Longevity does not mean health, and there is little evidence that the elderly who live longer today are healthier than their elders at the same age because people are increasingly at risk of chronic diseases, even the multimorbidity (2, 3). Multimorbidity, defined as the presence of two or more disease conditions, is increasingly becoming a public health problem (4). Life expectancy estimates are useful measures for decisions related to public health and primary health care. Evidence has confirmed that life expectancy is increasing, yet quality of life has not improved (5–7). In other words, healthy life years are increasingly lost due to multimorbidity, especially in the last years of life (8–11). Many previous studies have found an association between the living risk and the number of years of life lost due to multimorbidity. A healthy lifestyle could extend life expectancy by more than 8 years in Japan and over 10 years in Germany and the UK. Additionally, some cohort studies estimated the association between lifestyle and life expectancy and suggested that a low-risk lifestyle can increase life expectancy (12–16). Similarly, a population-based cohort study assessed the lifetime risks of developing cooccurring chronic diseases and quantified their multimorbidity, showing that multimorbidity started contributing to the loss of healthy life years beginning at age 45 (17).

However, the cause of the loss of healthy life years due to multimorbidity in the extended life years is still unclear, and there is no evidence for the influence of different living risks on the loss of healthy life years due to multimorbidity. In this study, we observed the incidence of multimorbidity during the follow-up period, adjusting for sex and area, and explored the association between the living risk and the healthy life years lost due to multimorbidity.

## Methods

### Data Source and Study Population

This research was conducted using data from the China Health and Retirement Longitudinal Study (CHARLS), a national cohort consisting of baseline measurements taken in 2011 with biennial follow-ups, including assessments of demographic variables, health status and function, health care and insurance, work, and retirement and pension. The CHARLS's baseline survey included 13,949 individuals who received all survey questions. A stratified (by per capita GDP of urban districts and rural counties) multistage (county/district-village/community-household) PPS random sampling strategy was adopted. A detailed description of the CHARLS was published previously (18, 19). For this study, the following were exclusion criteria: age under 45 years (*n* = 268); missing health-related data (*n* = 676); missing covariate data (*n* = 31); baseline comorbidity (*n* = 3,837); and death in 2011 (*n* = 46). A total of 9,091 observations were ultimately included (Supplementary Figure 1).

### Mortality

Age-sex-area-specific mortality data were obtained from the National Cause of Death Monitoring Dataset in 2018, a database generated by the nationally representative death cause monitoring system with 605 monitoring points across China. It can be obtained from the website http://ncncd.chinacdc.cn/jcysj/siyinjcx/syfxbg/202101/t20210118_223798.htm free of charge.

### Defining Multimorbidity

We identified 11 self-reported diseases and the time of multimorbidity by asking “When was the disease first diagnosed or known by yourself?”: cancer or malignant tumor; chronic lung disease; liver disease; heart problems; stroke; kidney disease; stomach or other digestive disease; emotional, nervous or psychiatric problems; memory-related disease; arthritis or rheumatism; and hip fracture. Participants with 2 or more of these conditions were classified as having multimorbidity, and we combined the self-reported diseases collected at the three previous time points to determine the final time with multimorbidity. Because hypertension, diabetes or high blood sugar, and dyslipidemia may cause metabolic disorders and lead to the occurrence of multimorbidity, these diseases previously considered in the definitions of multimorbidity were not included in this analysis.

### Assessments of Covariates and Health-Related Factors

All models were adjusted for sex, area (urban areas vs. rural areas), and age. Education was categorized into two levels: “having 9 years and less of education” (including illiterate, did not finish primary school, Sishu/home school, elementary school, middle school, high school, and vocational school) and “having over 9 years of education” (including 2-/3-year college/associated degree, 4-year-college/bachelor's degree, master's degree, and doctoral degree/Ph.D.). Smoking was categorized as having smoking experience (including having ever chewed tobacco, smoked a pipe, smoked self-rolled cigarettes, or smoked cigarettes/cigars) at the time of assessment or not having smoking experience. The participants were grouped as reported having drank alcoholic beverages (including beer, wine, or liquor) or not having drank in the past year. The body mass index (BMI) was calculated by the ratio of self-reported weight (kg) and the square of self-reported height (m^2^). We defined overweight or obesity as a BMI over 24.0 (kg/m^2^); otherwise, participants were categorized as not overweight or obese. Hypertension was defined as the participant being diagnosed with hypertension by a doctor. Having dyslipidemia was defined as the participant having been diagnosed with dyslipidemia (including elevation of low-density lipoprotein, triglycerides (TGs), and total cholesterol, or a low high density lipoprotein level) by a doctor. Diabetes was defined as the participant being diagnosed with diabetes or high blood sugar by a doctor.

### Statistical Analysis

We defined having over 9 years of education, not smoking, not drinking, not overweight or obesity, not having hypertension, not having dyslipidemia, or diabetes as the reference level for categorical variables. For each reference level, the participant received a score of 0 if he or she met the criterion and 1 otherwise. The sum of these seven health-related factors together gave a final living risk score ranging from 0 to 7, with higher scores indicating a less healthy lifestyle. Finally, we categorized participants into three groups based on the tertiles of the living risk scores: namely, low-risk, medium-risk, and high-risk.

First, we preliminarily described the characteristics of the data. Counts and percentages were reported for categorical variables [No. (%)], and continuous variables were summarized by the mean and SD (M ± SD). The chi-square test and Fisher's exact test were used to evaluate the statistical significance of the differences in baseline characteristics. Then, we used the Cox proportional hazards regression, with time since baseline assessment as the start of follow-up, to assess the association between living risk and multimorbidity. If no disease was present at baseline, the time to multimorbidity was derived as the difference between the time when the second disease occurred and the time when the first disease occurred. If one disease was present at baseline, the time to multimorbidity was derived as the difference between the time of second diagnosis and baseline. Hazard ratios (HRs) and corresponding 95% CIs (95% CI) were calculated, and the Schoenfeld's residuals were used to verify the proportional hazard assumption. Three incremental models were fitted: Model 1—unadjusted; Model 2—adjusted for age; and Model 3—adjusted for age, sex, and area (Supplementary Table 1). We estimated the prevalence of multimorbidity for each age group based on the probability non-multimorbidity of different age groups. We used the log rank method to verify the significance of multimorbidity in different living risk groups. Finally, we calculated the healthy life years lost caused by multimorbidity by the difference in life expectancy and healthy life expectancy through the Sullivan method.

We conducted two sensitivity analyses to assess the robustness of our results. First, we used a parametric Weibull model to assess the impact of different living risks on the prevalence of multimorbidity. In a second sensitivity analysis, the accelerated failure-time model (AFT) was used to explain the impact of different living risks on the duration of multimorbidity (further details are provided in Supplementary Table 1).

All tests used the 2-tailed tests; *P* < 0.05 was considered statistically significant. All analyses were performed using the Stata 15 SE, the SAS 9.4, and the R 3.6.0.

## Results

### Characteristics of the Study Participants

Among the 13,949 participants selected from the CHARLS baseline survey in 2011, 9,091 (51.35%) did not have multimorbidity and had complete data available at baseline. The mean age of participants without disease was 58.49 ± 9.73 years, and it was 59.55 ± 9.50 years for participants with one disease. Most participants with one disease were women (52.6%). Participants with lower education (98.71%), not drinking (67.61%), having hypertension (48.84%), having dyslipidemia (24.57%), and having diabetes (8.81%) were more likely to have one disease (*P* < 0.05). For the combined health-related factor score, participants in the low-risk, medium-risk, and high-risk groups had disease probabilities of 53.06, 28.82, and 18.13%, respectively. The participants in the high-risk group were more likely to have one disease (Table 1).

**Table 1:** Baseline characteristics of participants with or without disease.

	**Without disease (*n* = 5,152)**	**With one disease (*n* = 3,939)**	***P-*value**
Age (year)	58.49 ± 9.734	59.55 ± 9.50	**<0.001**
Sex			**0.013**
Male	2,564 (49.77)	1,867 (47.4)	
Female	2,588 (50.23)	2,072 (52.6)	
Area			**0.045**
Urban	462 (8.97)	313 (7.95)	
Rural	4,690 (91.03)	3,636 (92.31)	
Education			**0.001**
Over 9 years	112 (2.17)	51 (1.29)	
Nine years and less	5,040 (97.83)	3,888 (98.71)	
Smoking experiences			0.426
No	3,090 (59.98)	2,354 (59.76)	
Yes	2,062 (40.02)	1,585 (40.24)	
Drinking			**<0.001**
No	3,285 (63.76)	2,663 (67.61)	
Yes	1,867 (36.24)	1,276 (32.39)	
Overweight or obesity			0.361
No	3,086 (59.9)	2,344 (59.51)	
Yes	2,066 (40.1)	1,595 (40.49)	
Hypertension			**<0.001**
No	4,192 (81.37)	2,971 (75.43)	
Yes	960 (18.63)	968 (24.57)	
Dyslipidemia			**<0.001**
No	4,833 (93.81)	3,592 (91.19)	
Yes	319 (6.19)	347 (8.81)	
Diabetes			**0.006**
No	4,931 (95.71)	3,724 (94.54)	
Yes	221 (4.29)	215 (5.46)	
Living risks			**<0.001**
Low-risk	2,821 (54.76)	2,090 (53.06)	
Medium-risk	1,555 (30.18)	1,135 (28.81)	
High-risk	776 (15.06)	714 (18.13)	

*Bold P-values indicate statistical differences*.

*Over nine years of education: two-/three-year college/associated degree, four-year- college/bachelor's degree, master's degree and doctoral degree/Ph.D*.

*Overweight or obesity defined as their BMI being over 24.0 (kg/m2)*.

*Having hypertension, dyslipidemia or diabetes defined as having been diagnosed by doctor*.

*HR, hazard risk; CI, confidence interval*.

### Living Risk and the Probability Without Multimorbidity

**Figure 1:**
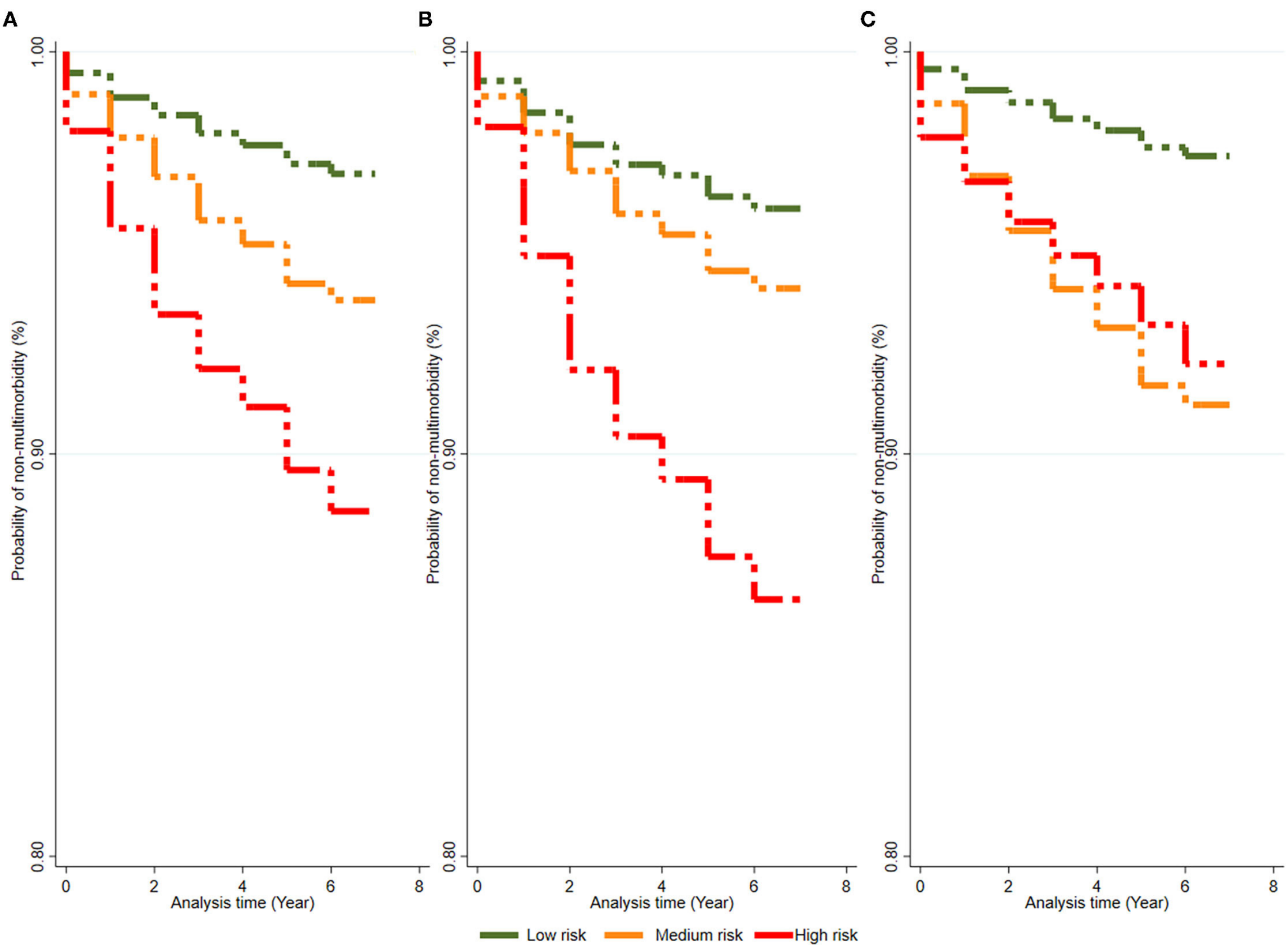
The change of probability without multimorbidity of people in seven-year-follow-up. **(A–C)** Represent the probability of non-multimorbidity in total population, males and females, respectively.

In all participants, the probability without multimorbidity declined continuously during the 7-year follow-up, and the probability of more healthy years with multimorbidity in the low-risk group was greater than that in the medium-risk group and high-risk group (Figure 1). A, B, and C represent the probability of no multimorbidity overall in all participants, in men, and in women, respectively. Overall, the probability of no multimorbidity decreased from 0.9947 to 0.9697 over the 7 years in the low-risk group, 0.9894–0.9382 in the medium-risk group, and 0.9803–0.8858 in the high-risk group. Compared with women (from 0.9787 to 0.9224), the probability of no multimorbidity for men in the high-risk group at the time of last assessment was lower (from 0.9813 to 0.8639). The rate without multimorbidity in the high-risk group in urban areas changed by 41.75% (Supplementary Figure 2). As the follow-up time increased, the likelihood of living without multimorbidity decreased. In contrast, the change over 7 years for the high-risk group in rural areas (7.81%) was not as large.

### Living Risks and Multimorbidity

**Figure 2:**
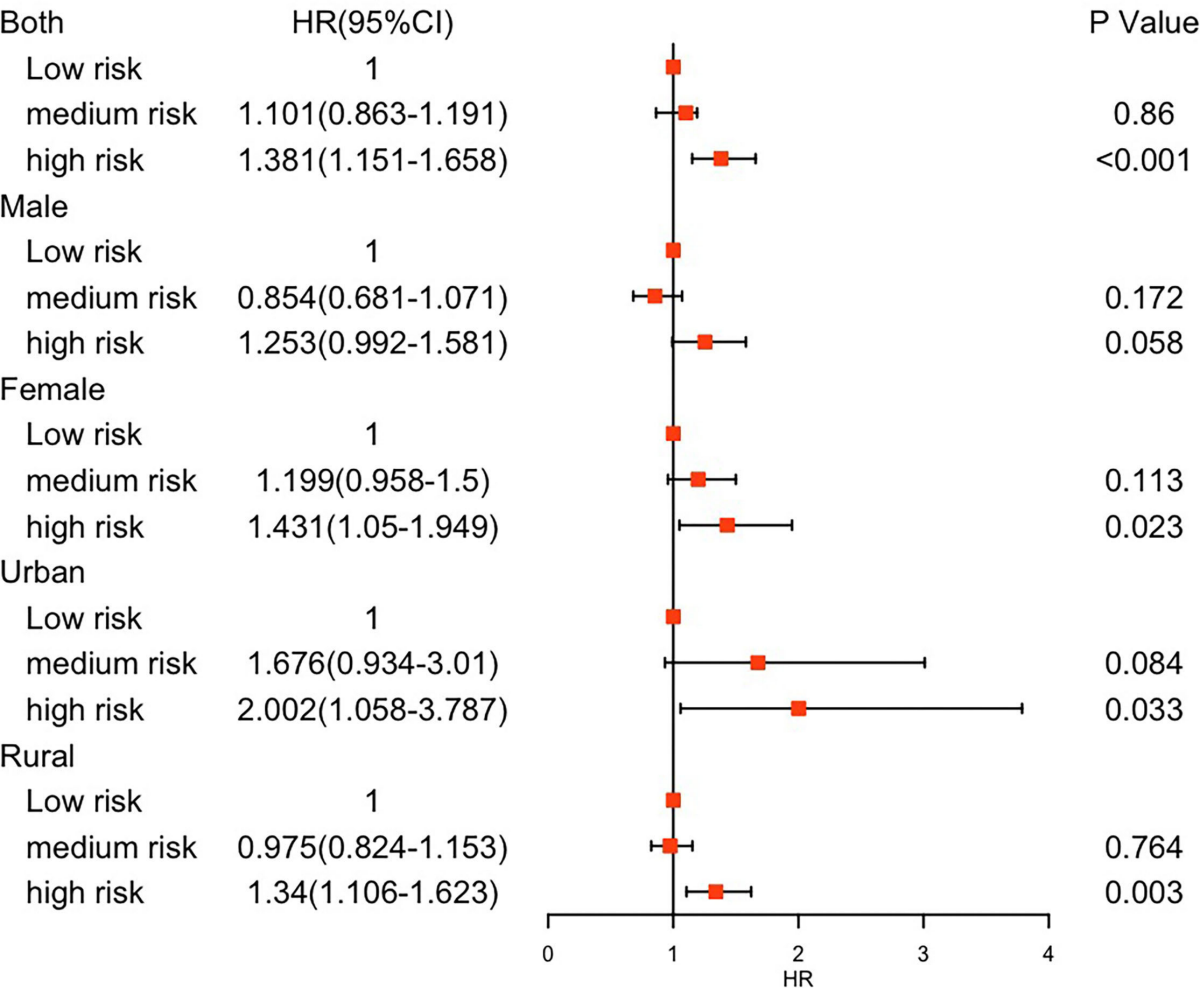
Hazard risks of multimorbidity by living risk group. HR, hazard risk; CI, confidence interval.

During a follow-up of 7 years, 908 multimorbidity cases were observed. The results for HR in the three incremental models are shown in Supplementary Table 2. Compared to the reference group (low-risk group), the adjusted HRs of multimorbidity were different for the other risk groups for both sex and area. HRs in the medium-risk group ranged from 0.854 (95% CI 0.681–1.071; *P* = 0.172) to 1.199 (0.958–1.500; *P* = 0.113) by sex and from 0.975 (95% CI 0.824–1.153; *P* = 0.764) to 1.676 (0.934–3.010; *P* = 0.084) by area. HRs in the high-risk group ranged from 1.253 (95% CI 0.992–1.581; *P* = 0.058) to 1.431 (1.05–1.949; *P* = 0.023) by sex and from 1.340 (95% CI 1.106–1.623; *P* = 0.003) to 2.002 (1.058–3.787; *P* = 0.033) by area (Figure 2).

### Healthy Life Years Lost Due to Multimorbidity

**Figure 3:**
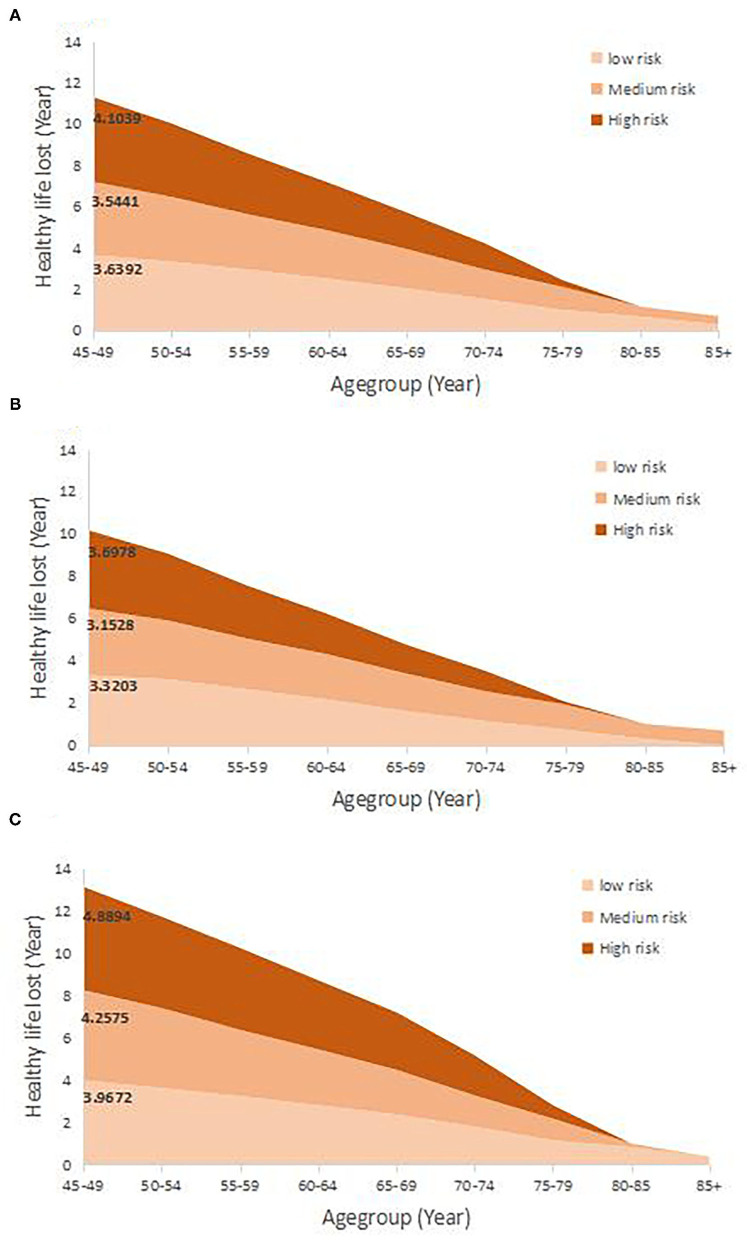
The healthy life years lost due to the multimorbidity in 7-year follow-up. **(A–C)** Represent the healthy life lost in total population, males and females, respectively.

Figure 3 shows the years of healthy life lost due to multimorbidity. A, B, and C represent high-risk group data for the situation in all participants, in men, and in women, respectively, and the healthy life years lost in urban areas and rural areas are shown in Supplementary Figure 3. The years of healthy life lost due to multimorbidity decreased as the level of living risk decreased. After adjusting for covariates, at age 45 in all participants, the life expectancy decreased by ~3.64 healthy life years, 3.54 healthy years, and 4.10 healthy life years in the low-risk, medium-risk, and high-risk groups, respectively. The years of healthy life lost in men (low-risk, medium-risk, and high-risk; 3.32, 3.15, and 3.70 years, respectively) was less than that in women (in low-risk, medium-risk, and high-risk; 3.97, 4.26, and 4.89 years, respectively) at age 45 regardless of the risk group. Except for in the low-risk group (2.61 years in urban areas and 3.70 years in rural areas), the years of healthy life lost in urban areas (3.87 years in the medium-risk group and 4.19 years in the high-risk group) were higher than those in rural areas (3.49 years in the medium-risk group and 4.07 years in the high-risk group). Participants who had multimorbidity starting at age 65 years had no significant changes in the healthy part of additional life years in the low-risk group. Regardless of sex and area, compared with that of low-risk group, the change in healthy life loss was lower for people with multimorbidity in the medium-risk group.

### The Association Between Health-Related Factors and the Hazard of Multimorbidity

**Figure 4:**
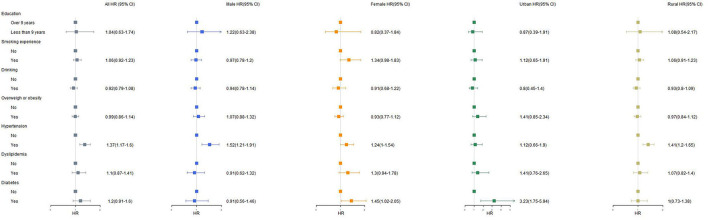
The association with health-related factors and multimorbidity. Over 9 years of education: 2-/3-year college/associated degree, 4-year-college/bachelor's degree, master's degree and doctoral degree/Ph.D. Overweight or obesity defined as their body mass index (BMI) being over 24.0 (kg/m^2^). Having hypertension, dyslipidemia or diabetes defined as having been diagnosed by doctor. HR, hazard risk; CI, confidence interval.

The associations between health-related factors and the hazard of multimorbidity are presented in Figure 4. A significant hazard risk was observed for hypertension: The probability of multimorbidity for participants with hypertension compared to that for those without hypertension was 1.5 times lower in males (95% CI: 1.21–1.91; *P* < 0.001) and also lower in women [HR = 1.24 (95% CI: 1–1.54, *P* = 0.05)]. In urban areas, people with diabetes had 3.2 times (95% CI: 1.75–5.94, *P* < 0.001) higher risk of multimorbidity than people without diabetes.

## Discussion

In this cohort study, we found that in participants with the lowest health-related factor score (low-risk group), the probability of no multimorbidity was lower than that of the medium-risk group. We found that for people with multimorbidity in the low-risk group at age 45, the average healthy life years was greater regardless of sex and area as compared to those in the high-risk group. The results are nearly consistent in multiple sensitivity analyses. These findings are relevant for public health and also have implications for individuals, as the data suggest that membership in the low-risk group is associated with fewer lost healthy life years due to multimorbidity.

To our knowledge, this is the first study to quantify the healthy life loss of the additional years of life caused by multiple diseases under different groups defined by living risks. In terms of the relationship between life years and multimorbidity, numerous studies have investigated whether the coexistence of diseases increases the loss of life expectancy. The results of a life table analysis found that living with multiple chronic conditions has a negative impact on life expectancy that is reduced by an average of 1.8 years with each additional chronic condition (20). A large cohort study observed that a low-risk lifestyle was associated with a longer life expectancy at age 50 for participants who were free of major chronic diseases (21). Many studies have also confirmed that healthy years account for fewer and fewer additional life years, which may be due to the existence of comorbidities of chronic diseases (6, 9, 22). Our results are consistent with these studies, and we confirmed that multimorbidity was associated with a loss of healthy life years. The loss of healthy life years due to multimorbidity differs between the sexes. Many studies have shown that women have longer survival times than men but are not healthier in the additional years. Our results also verify the conclusions of previous studies that showed that women have a higher probability of multimorbidity in different living risks than men. Compared to the high-risk group, membership in the low-risk group was associated with a gain of 0.46 additional healthy life years overall with multimorbidity and 0.38 healthy life years, 0.92 healthy life years, 1.58 healthy life years, and 0.38 healthy life years in men, women, urban areas, and rural areas, respectively. Membership in the low-risk group was not only associated with a decreased risk of incident multimorbidity but also improved the healthy survival years after diagnosis with those diseases (16, 23, 24).

Seven health-related factors were included in this analysis, including smoking, drinking, overweight or obesity, hypertension, dyslipidemia, and diabetes, as these factors are related to multimorbidity. Education plays an important role in improving healthy lifespan (25). A previous study emphasized that smoking, overweight or obesity, hypertension, and diabetes had an impact on healthy life expectancy (26–29). A European study indicated that people with behavior-related high-risk lifestyles have nearly 6 years lost due to multimorbidity (13). However, the health loss due to drinking is still controversial (10, 30, 31). The results of the factors in this study had similar outcomes. Drinking alcohol reduces the risk of multimorbidity, but there is no significant difference, which may be the reason why the probability of multimorbidity was lower in the medium-risk group than in the low-risk group. Hypertension and diabetes can cause multimorbidity and have been subjects of concern in many countries and regions (32, 33). Several recommendations for national policies and efforts need to be implemented to combat the further development of hypertension.

The findings from this study confirmed that an association exists between living risks and multimorbidity, adding to the observed association with healthy life years lost due to multimorbidity. However, this study has several limitations. First, we did not include physical activity as a health-related factor to better assess the robustness of the results because of the lack of availability of metabolic equivalent (MET)-min/week data in baseline measurements. However, a number of large cohort studies have verified the contribution of physical activity in reducing the probability of multimorbidity and improving health expectations (34–36). Second, the data on multimorbidity are all self-reported measures, which could underestimate the loss of healthy life years due to multimorbidity as a result of underreporting by participants. Then, with the popularization of health education in China and the health literacy of people has gradually improved, more and more people have begun to change their lifestyles. This study did not consider the impact of dynamic changes in health-related factors on healthy years of life lost due to multimorbidity, which may underestimate the extent of loss of healthy lifespan from multimorbidity. Finally, the date of diagnosis of chronic diseases is the date of recall of the respondent, which may give rise to a certain bias. At the same time, there is currently no accurate method to measure multiple morbidities (37). Our research includes the 11 most common chronic diseases and defines the concept of multimorbidity, and we also verify the reliability of the results through the other two methods. Unfortunately, we could not determine any causal relationship from our results due to the observational study design. Many studies have confirmed the relationship between different living risks and the life years lost due to multimorbidity (4, 20, 38).

## Data Availability Statement

Publicly available datasets were analyzed in this study. This data can be found here: https://charls.charlsdata.com/pages/data/111/zh-cn.html; http://ncncd.chinacdc.cn/jcysj/siyinjcx/syfxbg/202101/t20210118_223798.htm.

## Ethics Statement

The studies involving human participants were reviewed and approved by the National School of Development of Peking University. The patients/participants provided their written informed consent to participate in this study.

## Author Contributions

XM, QW, JC, WM, LR, YC, and ZW analyzed, interpreted the data regarding the disease, and the health-related factors. XG, XZ, and QM coordinated the study. XM was a major contributor in writing the manuscript. XM, JC, and WM revised the manuscript. QM and XZ edited the manuscript. All authors read and approved the final manuscript.

## Funding

This project was funded by the Project of Standardized test point of telemedicine information system. The funding body had no role in the design of the study and collection, analysis, and interpretation of data or in writing the manuscript.

## Conflict of Interest

All authors declare that the research was conducted in the absence of any commercial or financial relationships that could be construed as a potential conflict of interest.

## Publisher's Note

All claims expressed in this article are solely those of the authors and do not necessarily represent those of their affiliated organizations, or those of the publisher, the editors and the reviewers. Any product that may be evaluated in this article, or claim that may be made by its manufacturer, is not guaranteed or endorsed by the publisher.
