# Supplementary material for: Association Between Living Risk and Healthy Life Years Lost Due to Multimorbidity: Observations From the China Health and Retirement Longitudinal Study
Source: Front Med (Lausanne). 2022 Mar 17;9:831544. doi: 10.3389/fmed.2022.831544 (PMC8970175; doi:10.3389/fmed.2022.831544)
Supplement: Supplementary file 1 [file Data_Sheet_1.docx]

Supplementary Material

# Flow chart of this current study

**
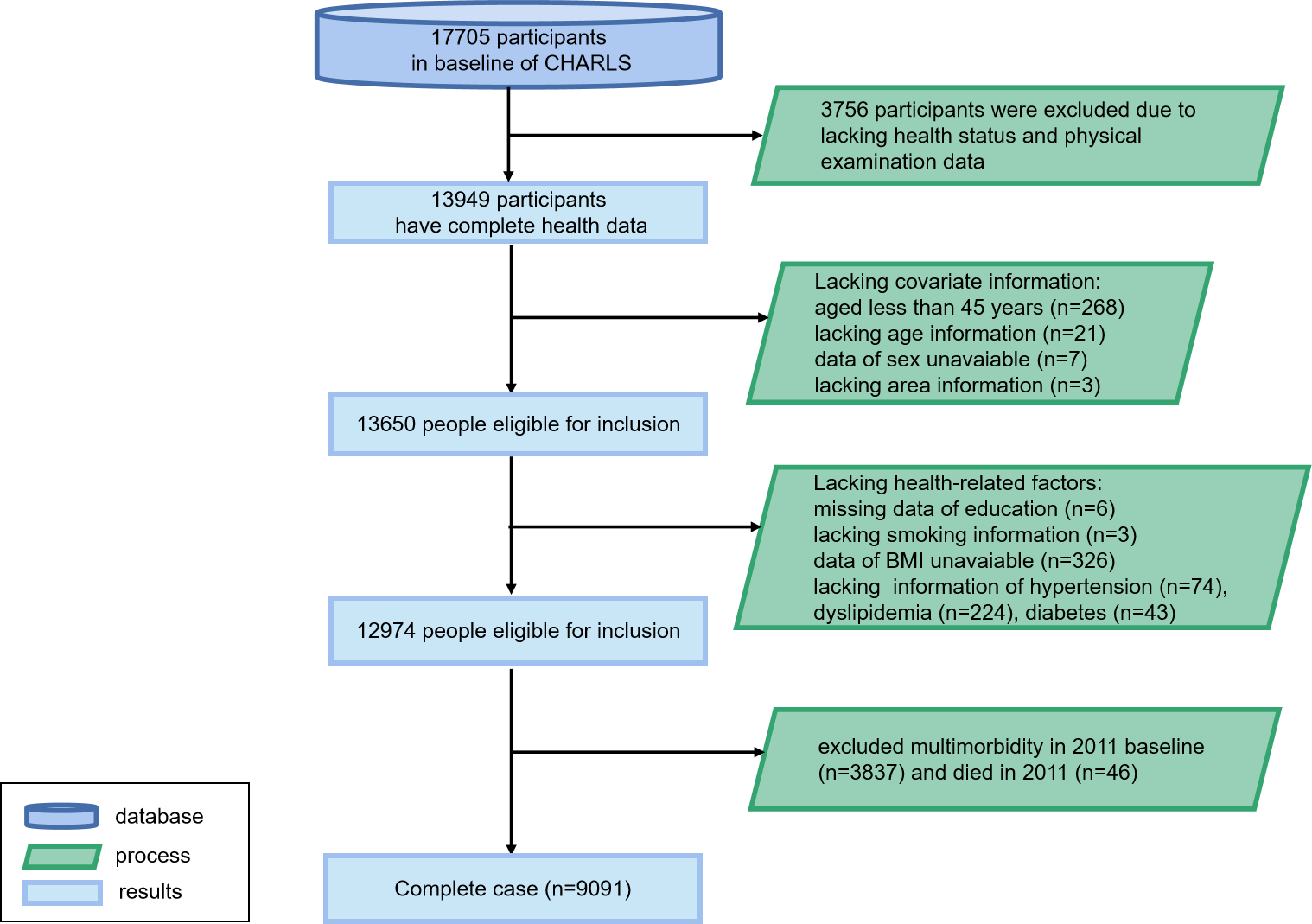
**

**eFigure 1 Flow chart of this current study**

# Summary of sensitivity analyses

The results of Weibull model shows that increasing age will rise the risk of multimorbidity, HR=1.013 (95%CI: 1.006-1.020, P<0.001). Compared with low living risk, medium living risk HR=1.016 (95%CI: 0.865-1.194, P=0.843) and high living risk living HR=1.013 (95%CI: 1.157-1.667, P<0.001) both have an effect on probability of mulitimorbidity. Accelerated Failure-time model verify the stability of the results again that age and high living risk have the contribution to the probability of multimorbidity.

eTable 1 Summary of sensitivity analyses

|  | **Weibull Model** | | | **Accelerated Failure-time model** | | |
| --- | --- | --- | --- | --- | --- | --- |
|  | **HR** | **P Value** | **95%CI** | **Coef.** | **P Value** | **95%CI** |
| Sex ^a^ | 1.087 | 0.251 | 0.943-1.254 | -0.069 | 0.295 | -0.198-0.06 |
| Age | 1.013 | <0.001 | 1.006-1.02 | -0.013 | <0.001 | -0.019--0.006 |
| Area ^b^ | 1.109 | 0.414 | 0.865-1.423 | -0.093 | 0.408 | -0.315-0.128 |
| Medium risk c | 1.016 | 0.843 | 0.865-1.194 | -0.008 | 0.908 | -0.153-0.136 |
| High risk ^c^ | 1.389 | <0.001 | 1.157-1.667 | -0.299 | 0.001 | -0.469--0.128 |
| _cons | 0.004 | <0.001 | 0.003-0.007 | 4.985 | <0.001 | 4.512-5.457 |
| /ln_p | 0.187 | <0.001 | - | 0.538 | <0.001 | - |

a Reference to male

b Reference to urban areas

c Reference to low living risk

# The results for HR in three incremental models

eTable 2 Association between living risks and multimorbidity

|  | **Model1** | |  | **Model2** | |  | **Model3** | |
| --- | --- | --- | --- | --- | --- | --- | --- | --- |
|  | **HR(95%CI)** | **P Value** |  | **HR(95%CI)** | **P Value** |  | **HR(95%CI)** | **P Value** |
| Low risk | 1 (reference) |  |  | 1 (reference) |  |  | 1 (reference) |  |
| Medium risk | 0.989  (0.849-1.15) | 0.886 |  | 0.984  (0.844-1.146) | 0.833 |  | 1.014  (0.863-1.191) | 0.868 |
| High risk | 1.132  (1.11-1.56) | 0.001 |  | 1.324  (1.116-1.570) | 0.001 |  | 1.381  (1.151-1.658) | 0.001 |

Model 1: unadjusted, Model 2: adjusted for age, Model 3:adjusted for age, sex and area

HR: hazard risk

CI: confidence interval

# The change of probability without multimorbidity of people in seven-year-follow-up in urban and rural areas

**
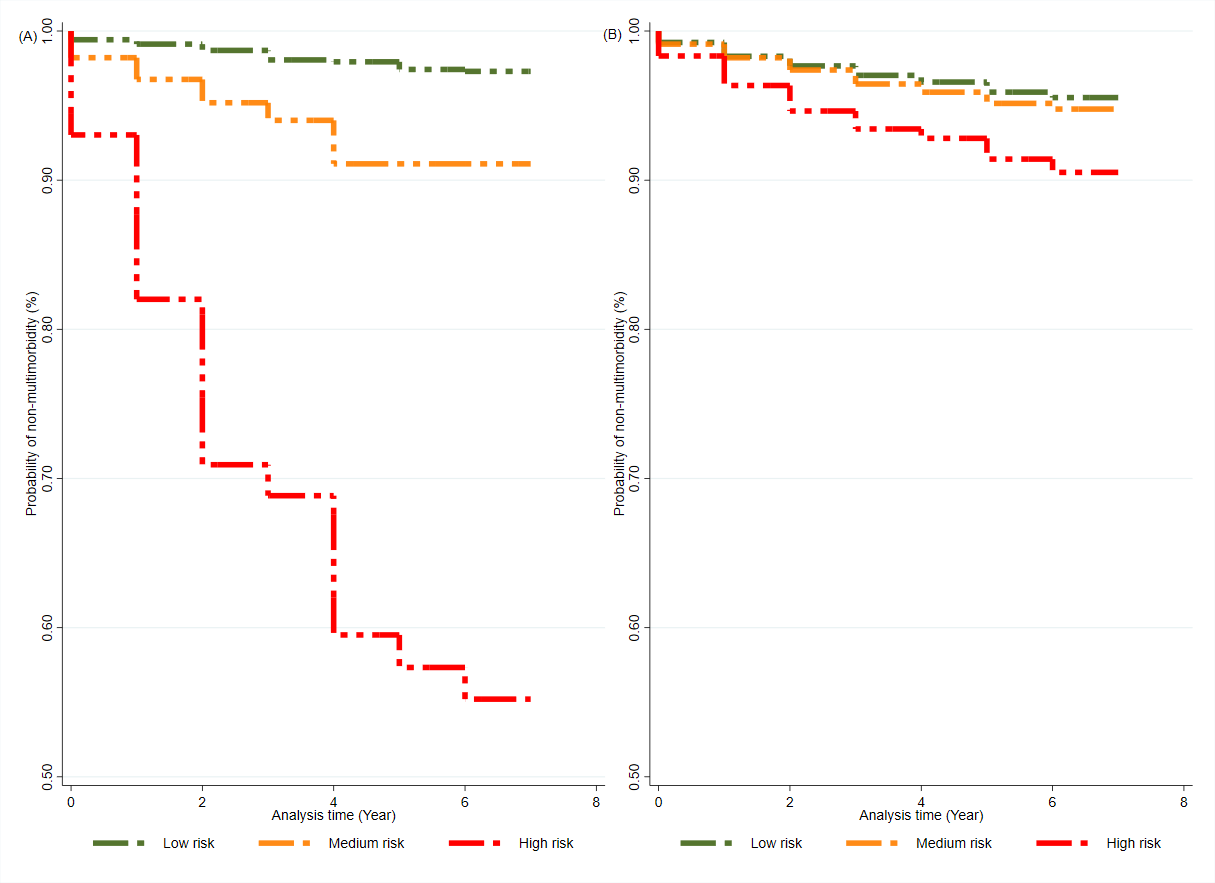
**

**eFigure 2 The change of probability without multimorbidity of people in seven-year-follow-up in urban and rural areas**

A to B represents the probability of people without multimorbidity in urban areas and rural areas, respectively.

# The healthy years of life lost in urban areas and rural areas

**
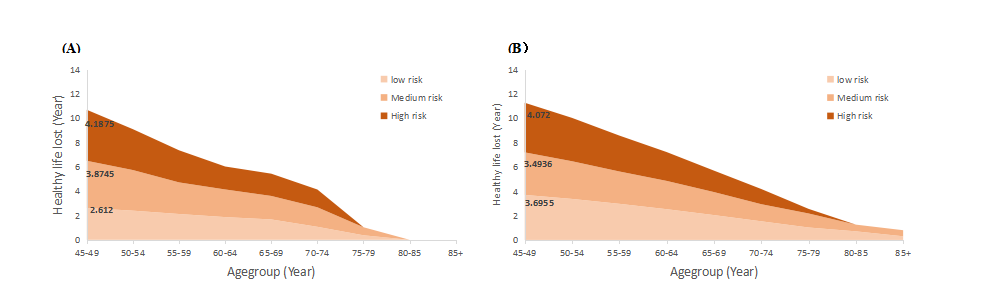
**

**eFigure 3 The healthy years of life lost in urban areas and rural areas**

A to B represents the healthy years of life lost in urban areas and rural areas, respectively.
